# Axonal Degeneration in Tauopathies: Disease Relevance and Underlying Mechanisms

**DOI:** 10.3389/fnins.2017.00572

**Published:** 2017-10-17

**Authors:** Andrew Kneynsberg, Benjamin Combs, Kyle Christensen, Gerardo Morfini, Nicholas M. Kanaan

**Affiliations:** ^1^Neuroscience Program, Michigan State University, East Lansing, MI, United States; ^2^Department of Translational Science and Molecular Medicine, College of Human Medicine, Michigan State University, Grand Rapids, MI, United States; ^3^Department of Anatomy and Cell Biology, University of Illinois at Chicago, Chicago, IL, United States; ^4^Hauenstein Neuroscience Center, Mercy Health Saint Mary's, Grand Rapids, MI, United States

**Keywords:** axonal transport, Alzheimer's disease (AD), chronic traumatic encephalopathy (CTE), pick's disease, progressive supranuclear palsy, corticobasal degeneration, neurodegeneration, animal models of tauopathies

## Abstract

Tauopathies are a diverse group of diseases featuring progressive dying-back neurodegeneration of specific neuronal populations in association with accumulation of abnormal forms of the microtubule-associated protein tau. It is well-established that the clinical symptoms characteristic of tauopathies correlate with deficits in synaptic function and neuritic connectivity early in the course of disease, but mechanisms underlying these critical pathogenic events are not fully understood. Biochemical *in vitro* evidence fueled the widespread notion that microtubule stabilization represents tau's primary biological role and that the marked atrophy of neurites observed in tauopathies results from loss of microtubule stability. However, this notion contrasts with the mild phenotype associated with tau deletion. Instead, an analysis of cellular hallmarks common to different tauopathies, including aberrant patterns of protein phosphorylation and early degeneration of axons, suggests that alterations in kinase-based signaling pathways and deficits in axonal transport (AT) associated with such alterations contribute to the loss of neuronal connectivity triggered by pathogenic forms of tau. Here, we review a body of literature providing evidence that axonal pathology represents an early and common pathogenic event among human tauopathies. Observations of axonal degeneration in animal models of specific tauopathies are discussed and similarities to human disease highlighted. Finally, we discuss potential mechanistic pathways other than microtubule destabilization by which disease-related forms of tau may promote axonopathy.

## Introduction

Proper brain function relies on appropriate connectivity between specific neuronal populations. An essential cellular process underlying such connectivity involves the generation and continued maintenance of molecular constituents within axons and dendrites (Conde and Caceres, 2009; Rasband, 2010). Maintenance of axons is particularly challenging in neurons because of their large size and complex biochemical heterogeneity of discrete functional compartments (i.e., nodes of Ranvier and synapses) within this major neuronal subdomain (Morfini et al., 2001). The axonal cytoskeleton features a polarized microtubule organization, a characteristic that allows for bidirectional transport of membrane-bounded organelles (MBOs) to and from the neuronal soma (Baas et al., 2016). Specialized intracellular transport events include the regulated delivery of MBOs from the soma to the pre- and post-synaptic compartments, the removal of old materials from these compartments, and the maintenance of neurotrophic support, among many others (Morfini et al., 2001). Long distance MBO trafficking events, collectively referred to as axonal transport (AT), are mainly powered by the kinesin and dynein superfamily of microtubule-based molecular motors (Black, 2016; Morfini et al., 2016). To date, a large body of genetic and experimental evidence indicates that maintenance of the unique cytoarchitecture and connectivity of neurons depends on appropriate functionality of major AT components, including microtubules, molecular motors, and protein kinases involved in their regulation (Gibbs et al., 2015; Morfini et al., 2016). Accordingly, genetic mutations in specific molecular motor protein subunits are implicated as potential causative factors for various neurodegenerative diseases (Morfini et al., 2009; Kanaan et al., 2013; Brady and Morfini, 2017).

Tauopathies comprise a heterogeneous group of diseases, including Alzheimer's disease (AD), frontal temporal dementia with parkinsonism linked to chromosome 17 (FTDP-17), chronic traumatic encephalopathy (CTE), progressive supranuclear palsy (PSP), corticobasal degeneration (CBD), and Pick's disease (PiD) (Arendt et al., 2016). Being originally limited to observations derived from post-mortem brains at advanced disease stages, much of the past research on tauopathies focused on mechanisms linking aberrant accumulation of filamentous tau aggregates to cognitive decline and neuronal cell death. However, the recent development of animal models of tauopathies facilitated the identification of much earlier pathogenic events. Detailed pathological analysis of these models indicates that disease-specific symptoms coincide with reductions in synaptic function and neuronal connectivity that long precede the loss of neurons (Morfini et al., 2009). Collectively, the available evidence indicates that neurons affected in tauopathies follow a dying back pattern of degeneration (Higuchi et al., 2002; Kanaan et al., 2013; Brady and Morfini, 2017). Based on this knowledge, interventions focused on maintenance of neuronal connectivity, rather than prevention of cell death, may represent a more pressing therapeutic need for tauopathies (Cheng et al., 2010; Lingor et al., 2012). Identification of disease-relevant mechanisms linking tau to axonal and neuritic degeneration may provide specific molecular targets to improve neuronal connectivity in human tauopathies (Kanaan et al., 2013).

## Evidence of axonopathy in human tauopathies

Degenerating axons follow a stereotypical set of morphological changes, typically initiated by enlargement of areas known as swellings or spheroid bodies (Raff et al., 2002). Concomitantly, axons undergo thinning between spheroids, ultimately displaying a beaded appearance as dystrophy progresses. This thinning process continues until the axon becomes fragmented, and eventually degraded (Zhou et al., 1998). Accordingly, the extent of these progressive morphological changes can be used to evaluate axonal degeneration *in vivo* and *in vitro* (Gatto et al., 2015; Kneynsberg et al., 2016). Demyelination often accompanies this process, highlighting the interdependence among oligodendrocytes and their myelinated axon tract (Barres et al., 1993).

Distinctive clinical and neuropathological features of specific tauopathies including AD (reviewed in Masters et al., 2015); FTDP-17, (reviewed in Ghetti et al., 2015); PSP, (Williams and Lees, 2009); CBD, (reviewed in Kouri et al., 2011); PiD, (Mckhann et al., 2001); and CTE, (reviewed in Kiernan et al., 2015) are reviewed elsewhere. Below, we provide a concise review of independent studies in human brains, which collectively provide strong evidence supporting the contention that axonal pathology represents an early and critical pathogenic event common to multiple human tauopathies (Table 1).

**Table 1:** Features of axonopathy in human tauopathies.

**Disease**	**Affected brain regions**	**Tau pathologies**	**Neuronal/glial**	**Synapse loss**	**Signs of axonal degeneration**	***In Vivo* imaging observations**
Alzheimer's Disease	Entorhinal cortex, hippocampus, cortex	Neurofibrillary tangles, neuropil threads, neuritic plaques	Primarily neuronal	Yes	Dystrophic axons, axonal swelling, demyelination	Progressive loss of white matter in regions displaying tau pathology correlated with clinical presentation (PET and DTI)
Frontal Temporal Dementia with Parkinsonism Linked to Chromosome 17	Frontal and temporal cortices	Varies by case but can include neurofibrillary tangles and glial pathologies resembling sporadic tauopathies	Both	Yes	Dystrophic axons, axonal swelling, demyelination	White matter loss in symptomatic and asymptomatic carriers of FTDP-17 mutations (PET and DTI)
Chronic Traumatic Encephalopathy	Frontal and temporal cortices, hippocampus	Neurofibrillary tangles, astrocytic plaques, coiled bodies, neuritic threads	Both	Yes	Dystrophic axons, axonal injuries following head injury	White matter abnormalities observed in some athletes after mild traumatic brain injury (DTI)
Progressive Supranuclear Palsy	Basal ganglia, internal capsule, and thalamic fasciculus	Neurofibrillary tangles, globose tangles, tufted astrocytes, coiled bodies	Both	Yes	Dystrophic axons, axonal spheroids, demyelination	White matter loss corresponding to disease severity and symptomatic presentation (DTI)
Corticobasal Degeneration	Frontal and parietal cortices	Astrocytic plaques, coiled bodies, globose tangles, neuritic threads	Both	Yes	Dystrophic axons, swollen terminals, demyelination	White matter loss corresponding to clinical presentations (DTI and MRI)
Pick's Disease	Frontal, temporal, and parietal lobes; hippocampus	Pick bodies, neuropil threads, ramified astrocytes	Neuronal >glial	Yes	Varied thinning and thickening of axons, demyelination	Severe atrophy of cortical white matter (MRI)

### Alzheimer's disease (AD)

The strong focus on tau lesions in AD yielded a large body of data establishing axonal degeneration as a prominent neuropathological hallmark of this disease (Kanaan et al., 2013). Tau inclusions within dystrophic neurites, known as neuropil threads, are a robust neuropathological feature of AD brains that appear before neurofibrillary tangles (NFTs) form in neuronal somata (Kowall and Kosik, 1987; Ghoshal et al., 2002). Immunohistochemical evidence demonstrates that pathological forms of tau known to inhibit AT accumulate in neuropil threads, suggesting localized disruption of cellular processes critical for axonal maintenance in AD, including AT (Kanaan et al., 2011; Combs et al., 2016a). Observations of abnormal vesicle accumulations within dystrophic neurites of AD brains provide further support for this notion (Praprotnik et al., 1996; Dessi et al., 1997).

Consistent with AT deficits in AD, results from ultrastructural and immunohistochemical analyses of AD brains reveal a clear correlation between loss of synapses and the manifestation of cognitive deficits (Dekosky and Scheff, 1990; Terry et al., 1991). Highlighting the pathological relevance of these findings, brain imaging studies in living AD patients document significant atrophy of axon-rich white matter structures, suggesting that axonal degeneration represents a prominent feature of the disease. Brain regions affected in patients with mild cognitive impairment, a well-established prodromal AD stage, mainly include axonal projections in the perforant pathway and cortical regions (Stoub et al., 2006; Huang and Auchus, 2007), where axon demyelination also occurs (Sjobeck and Englund, 2003; Sjobeck et al., 2005; Ihara et al., 2010). In more advanced AD cases, white matter atrophy extends to the corpus callosum, where the degree of atrophy directly correlates with cognitive decline (Vermersch et al., 1996; Hampel et al., 1998). Diffuse tensor imaging (DTI) techniques quantitatively measure water diffusion within cellular structures to identify microstructural changes in white matter that may not be evident with other imaging techniques (Meerschaert et al., 2016). Significantly, results from DTI studies show alterations in specific axonal tracts connecting association cortices, while others involved with motor or visual functions are largely spared. Again, the extent to which these tracts are affected displays a close correlation with the degree of cognitive decline (Bozzali et al., 2002).

### Frontotemporal dementia with parkinsonism linked to chromosome 17 (FTDP-17)

FTDP-17 represents a subgroup of inherited early-onset tauopathies resulting from mutations in the gene encoding tau. The discovery of FTDP-17 tau mutations demonstrated that tau dysfunction alone suffices to cause neuronal dysfunction and degeneration (Foster et al., 1997; Hutton et al., 1998), and axonal pathology is seen in FTDP-17 brains. Specifically, positron emission tomographic (PET) imaging studies in FTDP-17 patients show the accumulation of tau aggregates in white matter areas (Wszolek et al., 1992). Interestingly, brain imaging studies found white matter alterations in pre-symptomatic disease stages (Rohrer et al., 2010; Dopper et al., 2014). Similar to neuropil threads in AD, mutant tau aggregates localize within dystrophic axons of FTDP-17 brains (Delisle et al., 1999; Murrell et al., 1999; Lippa et al., 2000; Kouri et al., 2014). As discussed below, animal models of specific FTDP-17 variants largely recapitulate the axonopathy phenotype observed in affected human brains.

### Chronic traumatic encephalopathy (CTE)

CTE is a neurodegenerative disease associated with repetitive subconcussive and mild traumatic brain injuries (reviewed in Blennow et al., 2016). Focal areas of neuronal and glial tau inclusions at the depths of cortical sulci and in perivascular regions are pathognomonic CTE lesions found in the frontal and temporal cortices, as well as the hippocampus (Mckee et al., 2012). Symptoms of diffuse axonal injuries, including axonal swellings, unregulated calcium influx, and cytoskeletal abnormalities are evident within the first 24 h after concussion and may persist for weeks (Blumbergs et al., 1994; Maxwell et al., 1995; Giza and Hovda, 2001). Tau-positive neuropil threads also are a prominent neuropathological feature of CTE and cognitive decline correlates with axonal atrophy in subcortical white matter (Tokuda et al., 1991; Kraus et al., 2007; Mckee et al., 2009). Pathological forms of tau, identified by conformation-dependent tau antibodies, were recently found to localize within axons of cortical white matter and neuropil threads in the cholinergic basal forebrain (Kanaan et al., 2016; Mufson et al., 2016). Using a PET tracer, protein aggregates, likely made up of tau, were identified within white matter tracts of former professional football players suspected of having CTE (Barrio et al., 2015). Membrane-associated proteins, including amyloid precursor protein, accumulate in axons after traumatic brain injuries, suggesting AT disruption may play a role in CTE pathogenesis (Uryu et al., 2007). Additionally, a multitude of DTI studies have identified white matter changes in athletes at risk for concussions or repetitive subconcussive impacts from several sports, including boxing, football, soccer, ice hockey, as well as in veterans exposed to blast trauma (Zhang et al., 2003; Koerte et al., 2012a,b; McAllister et al., 2014; Petrie et al., 2014).

### Progressive supranuclear palsy (PSP)

In PSP, tau aggregates are observed in both neurons (NFTs and globose tangles) and glial cells (tufted astrocytes and coiled bodies) (Pollock et al., 1986; Hauw et al., 1994). The presence of neuropil threads in PSP indicates the presence pathological tau in axons, particularly in the basal ganglia, internal capsule, and thalamic fasciculus (Hauw et al., 1990; Dickson, 1999). Additionally, axonal spheroids are found in subcortical white matter, the putamen, globus pallidus, and the subthalamic nucleus (Probst et al., 1988; Ahmed et al., 2008). Extending these findings, DTI studies in early PSP cases found evidence of white matter degeneration within the pons, substantia nigra, cerebellar peduncles, and corpus callosum and the degree of atrophy in some of these regions correlates to disease severity and onset of symptoms (Padovani et al., 2006; Knake et al., 2010; Whitwell et al., 2011; Zhang et al., 2016). Interestingly, PSP is characterized by abundant tau lesions termed coiled bodies within oligodendrocytes, the cells responsible for generating myelin sheaths in the central nervous system. Demyelination is particularly evident in white matter tracts of PSP-affected brains, directly correlating with tau burden in the superior cerebellar peduncle and red nucleus. These findings suggest that tau-induced oligodendrocyte dysfunction could indirectly contribute to the axonal degeneration phenotype observed in PSP (Ishizawa et al., 2000).

### Corticobasal degeneration (CBD)

**Figure 1:**
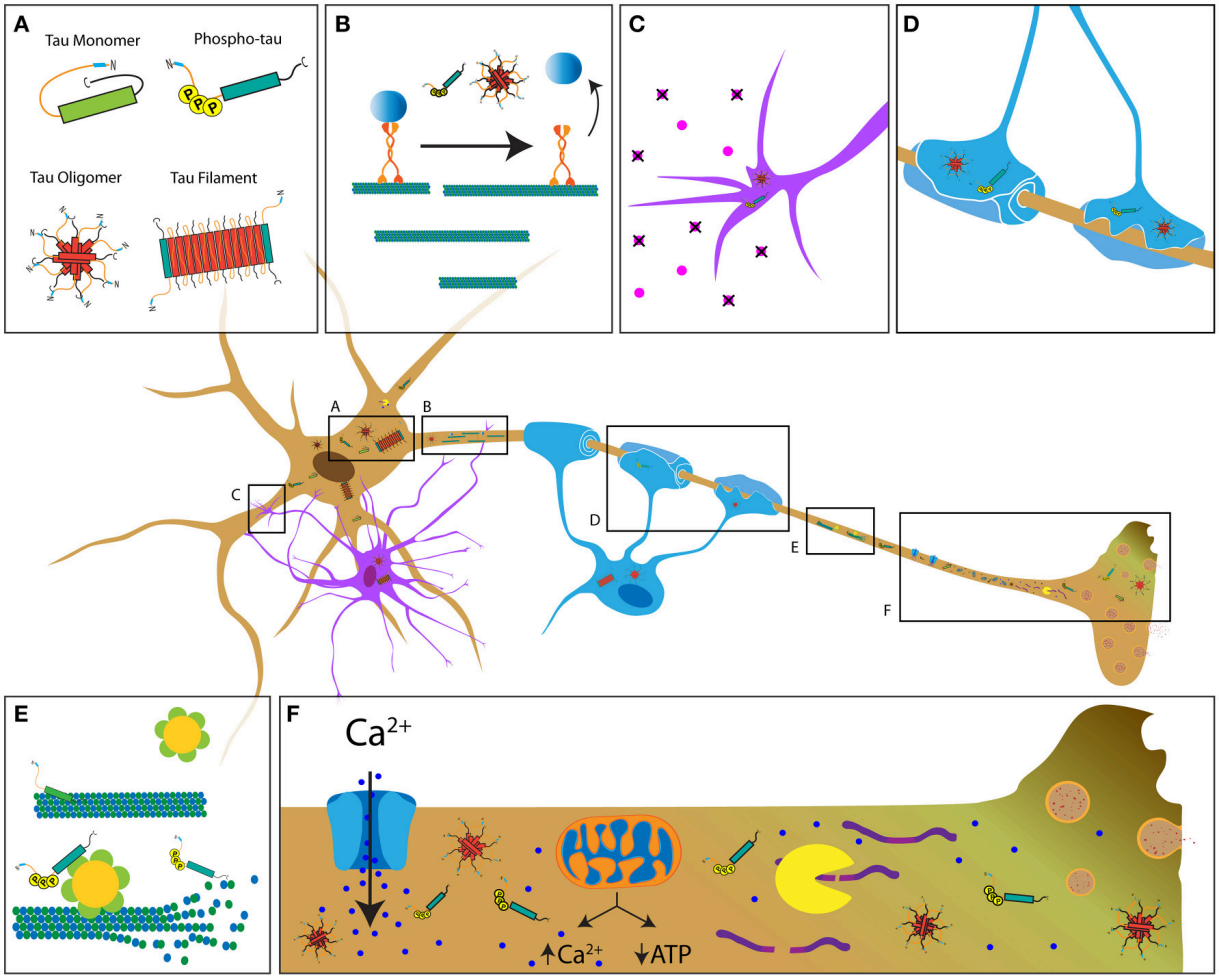
Potential mechanisms contributing to tau-induced axonal degeneration in tauopathies. **(A)** Pathological forms of tau include phosphorylated tau, tau oligomers, and tau filaments, all of which feature increased exposure of the PAD (blue region of tau) (Kanaan et al., 2011). In contrast, this domain is hidden in soluble tau monomers, which feature a paperclip conformation (Jeganathan et al., 2008). Depending on the specific tauopathy, pathological tau is present in neurons (brown cell), astrocytes (purple cell, **C**) and oligodendrocytes (blue cell, **D**). **(B)** Pathological conformations of tau impair axonal transport through activation of a phosphotransferase-based signaling pathway that promotes detachment of transported cargoes from the motor protein conventional kinesin. **(C,D)** Accumulation of pathological tau may also interfere with cellular processes involved in trophic support by astrocytes and oligodendrocytes, ultimately resulting in axonal dysfunction and demyelination. **(E)** Disease-related modifications of tau (e.g., phosphorylation) that reduce its binding to microtubules may promote aberrant katanin-mediated microtubule severing. **(F)** Disease-related forms of tau can alter Ca^2+^ homeostasis through various mechanisms, including abnormal modulation of ion channel activity, the endoplasmic reticulum and/or mitochondrial Ca^2+^ buffering. Enhanced Ca^2+^ levels may in turn increase calpain activity, leading to abnormal cleavage of cytoskeletal proteins. Together, these mechanisms represent the multifaceted pathways by which tau that likely contributes to axonopathy.

The characteristic tau pathology of CBD includes astrocytic plaques, occasional coiled bodies, and neuronal globose inclusions that are primarily found in the frontal and parietal lobes of the cerebral cortex, the cerebellum, and substantia nigra (Rebeiz et al., 1968; Feany and Dickson, 1995). Neuropil thread pathology is prominent in cortical gray and white matter, the subthalamic nucleus, and the striatum of CBD brains (Ikeda et al., 1994; Dickson, 1999). MRI and DTI-based studies find significantly greater white matter abnormalities in CBD (Doi et al., 1999; Zhang et al., 2016). Additional studies identified pathological changes in specific hand sensorimotor fiber tracts in patients who manifested limb apraxia at early CBD stages (Borroni et al., 2008), furthering the linkage between axonal degeneration and specific symptomatic outcomes. The extensive astroglial lesions in CBD and other tauopathies suggest that pathological forms of tau may affect astrocyte-specific functions critical to neuronal health, including sustained trophic support (Figure 1C; Kahlson and Colodner, 2015).

### Pick's disease (PiD)

Pick's disease (PiD) is characterized by severe atrophy of the frontal, temporal, and parietal lobes. Cytoplasmic neuronal tau inclusions known as Pick bodies represent the major neuropathological hallmark of PiD, and these are typically found in the dentate gyrus of the hippocampus and frontal and temporal cortices (Pollock et al., 1986; Probst et al., 1996). MRI studies of Pick's disease cases document severe atrophy of cortical white matter (Wang et al., 2006; Yamakawa et al., 2006). Microscopic evaluation of post-mortem brain tissue reveals that axons surrounding the dendrites of neurons in the affected brain regions (e.g., polymorphic layer of the dentate gyrus) often display pathological tau, in addition, glial tau pathology is observed (Cochran et al., 1994; Probst et al., 1996). Neuritic threads and spheroids are observed in mossy fibers projecting to the dentate nucleus along with abnormal tau in cerebellar white matter and other axons in PiD brains (Probst et al., 1996; Braak et al., 1999). A marked loss of myelinated axons is observed in subcortical white matter (Dickson, 1998) as well as perforant pathway synaptic loss (Lippa, 2004). Accumulation of AT-related molecules in Pick bodies (e.g., kinesin and synpatophysin) suggests AT is impaired in PiD (Nakamura et al., 1994).

Collectively, results from ultrastructural, immunohistochemical, and brain imaging-based studies strongly implicate axonopathy and the consequent neural disconnection as a pathogenic component common to all tauopathies. Localization of pathological forms of tau within dystrophic axons in each tauopathy further suggests alterations in one or more cellular processes critical for axonal maintenance, including AT (Morfini et al., 2002a; Kanaan et al., 2013).

## Animal models of tauopathies

Models of tauopathy using the expression of specific tau variants include transgenic mice and viral vector-mediated gene delivery of tau-encoding cDNA constructs in rodent brains, among others (Ballatore et al., 2007; LaFerla and Green, 2012; Combs et al., 2016b). As discussed below, these animal models exhibit characteristics reminiscent of those seen in human tauopathies, further supporting a role of axonopathy in the disease process.

Several transgenic tauopathy models were produced over the years, which replicate selected cardinal features of human tauopathies (Gotz et al., 2007). The early animal models are based on expression of wild-type human 4R (Gotz et al., 1995) and 3R (Brion et al., 1999) tau variants and these mice show only mild phenotypes. However, tau transgenic mice with stronger gene promoters produce a clear neurodegenerative phenotype and many of these transgenic models (e.g., the T44 and ALZ17 lines) feature tau pathology mainly in the brainstem and spinal cord (Ishihara et al., 1999; Spittaels et al., 1999; Probst et al., 2000). Axonopathy in these models manifests in the form of axonal spheroids, axonal neurofilament- and tau-immunoreactive inclusions (Spittaels et al., 1999). Expanding on these early models, the 8c transgenic mouse line expresses a human tau gene cassette that produces all six human tau isoforms and these mice display signs of axonopathy including axonal swellings and spheroids, but do not develop NFTs (Duff et al., 2000). The 8c line was later crossed with a tau knockout line to produce mice that exclusively express human tau isoforms in the absence of endogenous mouse tau (Tucker et al., 2001). Interestingly, mice resulting from this cross show early accumulation of pathological tau within axons (e.g., CP13 positive phospho-tau), before such changes are detected in neuronal somata (Andorfer et al., 2003). Thus, overexpression of exogenous human tau alone suffices to cause axonopathy, which appears inconsistent with microtubule destabilization playing a role in tauopathies. Instead, these observations suggest that exogenous transgenic tau promotes a toxic gain of function that affects the maintenance of axons (Kanaan et al., 2011).

After the discovery of autosomal dominant tau mutations in FTDP-17 families (Hutton et al., 1998), several groups generated transgenic mice expressing FTDP-17-related mutant forms of tau. Unlike wild-type tau expressing models, these mice successfully recapitulate NFT-like inclusions typical of tauopathies (Lewis et al., 2000; Gotz et al., 2001; Allen et al., 2002). P301L tau remains the most commonly studied FTDP-17 mutation (Dumanchin et al., 1998; Hutton et al., 1998; Rizzu et al., 1999), and several transgenic mouse models are based on expression of this mutant form of tau. JNPL3 transgenic mice for example, feature expression of 4R/0N P301L tau under control of the mouse prion promoter (Lewis et al., 2000). Interestingly, these mice feature not only NFT-like and pre-tangle tau inclusions, but also axonal spheroids and degenerated myelinated axonal tracts, predominantly in the spinal cord (Lewis et al., 2000; Lin et al., 2003, 2005). In rTg4510 mice, the P301L tau transgene is downstream of a tetracycline operon-responsive element (TRE), and they express a tetracycline-controlled transactivator (tTA) that is under control of the Ca^2+^-calmodulin kinase II-α (CaMKIIα) promoter, which provides relatively selective expression of tau in forebrain neurons (Mayford et al., 1996; Santacruz et al., 2005; Spires et al., 2006). Accordingly, mutant tau expression can be suppressed through doxycycline treatment, thus providing spatial and temporal control of tau expression (Santacruz et al., 2005). Interestingly, signs of axonopathy in the rTg4510 model are comparable to those in JNPL3 mice, including swollen axons, NFT-like inclusions, myelin loss, and accumulation of mitochondria in swollen axons (Ludvigson et al., 2011). The rTgTauEC model uses a similar Tet-Off system as the rTg4510 model. However, tTA expression is controlled by the neuropsin promoter (de Calignon et al., 2012), which restricts mutant tau expression to a subset of entorhinal cortex (EC) neurons (Yasuda and Mayford, 2006). In this model, initial signs of axonopathy include strong Alz50 immunoreactivity [Alz50 is an antibody that recognizes a pathological tau conformation (Carmel et al., 1996)] within axonal terminals of the outer two-thirds molecular layer of the dentate gyrus, followed by age-dependent axonal degeneration and loss of hippocampal neurons (de Calignon et al., 2012). Microglia activation and astrogliosis is observed in rTgTauEC mice, suggesting that glial dysfunction might exacerbate axonopathy in this model. Additionally, the rTgTauEC model shows spread of pathological tau through interconnected neurons, suggesting transsynaptic release of misfolded or aggregated tau into the synaptic cleft and uptake by downstream neurons (de Calignon et al., 2012). Based on these observations, it is reasonable to posit that synaptic and axonal degeneration may not only be a primary source of neuronal disconnection, but also promote the release and spread of pathological tau species through synaptically connected networks (reviewed in Walsh and Selkoe, 2016).

Glial contributions to axonopathy are an active area of research, and models that selectively overexpress tau in glial cells are valuable models of tauopathies. One such model expresses 4R/1N P301L tau specifically in oligodendrocytes, achieved by controlling expression through the 2′,3′-cyclic nucleotide 3′ phosphodiesterase promoter. These mice displayed oligodendrocyte tau inclusions, AT deficits that preceded axonal degeneration, and deficits in myelination (Higuchi et al., 2005). This model highlights the importance of oligodendrocytes on axonal maintenance because tau inclusions appear to promote deficits in oligodendrocyte function that in turn indirectly contribute to axonopathy (Figure 1D; Chin and Goldman, 1996; Dickson et al., 1996).

Viral vector-mediated gene delivery facilitates the introduction of exogenous genes with extensive spatial and temporal control over its expression. In wild-type mice, injection of recombinant adeno-associated viral (rAAV) vectors encoding P301L tau into the EC (Siman et al., 2013, 2015) or the hippocampus (Jaworski et al., 2009, 2011) results in axonal degeneration. Injected mice display accumulation of tau phosphorylated at the AT8 epitope within synapses, degeneration of axons in the performant pathway and spread of P301L tau into the dentate gyrus (Siman et al., 2013). Similar to some transgenic tau models, rAAV-based tauopathy models also display reactive gliosis around degenerating axons, which may contribute to axonal pathology (Siman et al., 2013, 2015). Furthermore, injection of an rAAV vector encoding pseudophosphorylated AT8 tau in the EC led to early signs of axonopathy, including the development of spheroids and a swollen morphology (Combs et al., 2016b). Similarly, lentiviral vectors encoding wild-type or P301L tau induce markers of axonopathy when injected in the rat hippocampus, that includes a time-dependent increase in swollen, fragmented, and dystrophic axons, as well as microglial activation (Caillierez et al., 2013; Hebron et al., 2014).

Collectively, data from various animal models of tauopathy reveal axonal degeneration as a prominent pathological feature, and further suggest there is a contribution to this phenotype from glial cells. Based on their resemblance to this specific aspect of human tauopathies, these animal models may help define mechanisms and specific molecular components mediating the toxic effect of pathogenic tau on axonal maintenance and function.

## Pathogenic mechanisms linking abnormal tau to axonopathy

The initial discovery of tau protein was based on biochemical procedures that revealed its ability to associate with purified microtubules and modulate their dynamic growth behavior *in vitro* (Weingarten et al., 1975; Cleveland et al., 1977a,b). These biochemical properties of tau quickly led to its designation as a microtubule-associated protein, and the suggestion that microtubule stabilization represents tau's primary functional role in neurons (Weingarten et al., 1975; Cleveland et al., 1977a,b). Several subsequent reports document various effects of tau on microtubule dynamics in cultured cell lines (reviewed in Feinstein and Wilson, 2005). By extension, microtubule destabilization resulting from loss of tau function is widely believed to represent a major mechanism underlying neuronal dysfunction and degeneration in human tauopathies (Guo et al., 2017). However, direct experimental evidence that tau is required for the maintenance of microtubule stability in cultured primary neurons or in neurons *in vivo* has yet to be provided.

Collectively, data from the available four transgenic tau knockout mouse lines (reviewed in Ke et al., 2012) reveal little to no effect on behavior, cognition, and neuropathology associated with germline removal of the tau gene (Harada et al., 1994; Dawson et al., 2001; Tucker et al., 2001; Fujio et al., 2007; Morris et al., 2011; van Hummel et al., 2016). These findings are inconsistent with a critical role for tau in sustaining microtubule stabilization *in vivo*. Furthermore, axonal development is normal in cultured primary neurons obtained from some tau knockout mice (Harada et al., 1994; Takei et al., 2000). Deleterious effects of removing tau documented so far are limited to mild behavioral and parkinsonian-like deficiencies with advancing age in a couple of mouse lines (Ikegami et al., 2000; Lei et al., 2014), alterations in microtubule density limited to small caliber cerebellar parallel fibers in one model (Harada et al., 1994), and delayed neurite development in cultured neurons from another (Dawson et al., 2001). Additional experiments using primary cultured neurons show that immunodepletion of axonal tau does not lead to appreciable alterations in axonal microtubule content or dynamics (Tint et al., 1998); thus, extending findings from tau knockout mice. Tau-independent mechanisms for microtubule stabilization, including post-translational modifications of tubulin and the existence of additional microtubule-associated proteins, could explain the marginal impact of tau deletion on microtubule stability (Song and Brady, 2015; Matamoros and Baas, 2016).

Available experimental evidence suggests that tau performs functions other than microtubule stabilization in neurons. Numerous studies suggest a role for tau in multiple cellular processes and compartments, some of which may not include microtubule interactions. The functional repertoire of tau includes microtubule-actin cytoskeleton interactions (Selden and Pollard, 1986; Liu et al., 1999), regulation of AT (LaPointe et al., 2009; Kanaan et al., 2011), nuclear and nucleolar functions (Papasozomenos and Binder, 1987; Loomis et al., 1990; Sultan et al., 2011), end-binding protein regulation (Ramirez-Rios et al., 2016), cytoskeleton-plasma membrane interactions (Brandt et al., 1995), RNA-binding protein and stress granule regulation (Vanderweyde et al., 2016), as well as scaffolding of phosphotransferases (Lee et al., 1998; Liao et al., 1998; Ittner et al., 2010; Kanaan et al., 2013). Supporting this later role, a number of protein kinases and phosphatases reportedly interact with tau including PP1 (Liao et al., 1998), GSK3β (Sun et al., 2002), Cdk5 (Li et al., 2006), Fyn (Lee et al., 1998), and others (Liu et al., 2016). Together, these reports support a role for tau as a scaffolding protein that targets phosphotransferases to microtubules; thus, facilitating their interaction with specific microtubule-associated protein substrates (e.g., motor proteins) (reviewed in Morfini et al., 2002b; Kanaan et al., 2013). Experimental evidence also suggests such a role may extend to the dendritic compartment, where tau appears to regulate localization and activity of the tyrosine protein kinase Fyn (Xia et al., 2015; Lau et al., 2016). Collectively, the available evidence supports a broader functional repertoire for tau unrelated to microtubule stabilization, providing clues to the potential mechanisms by which pathogenic forms of tau may promote axonopathy (Figure 1).

The dependence of axons on proper anterograde and retrograde AT is evident because mutations in selected conventional kinesins or cytoplasmic dynein subunits suffice to promote dying-back degeneration of neurons (Morfini et al., 2009). Providing an explanation for the axonal degeneration phenotype observed in tauopathies, multiple independent studies documented deficits in AT triggered by pathogenic forms of tau, including oligomeric tau (Higuchi et al., 2005; Gotz et al., 2006; Cox et al., 2016; Swanson et al., 2017). Several mechanisms are proposed to mediate this toxic effect. The early observation that tau overexpression leads to alterations in intracellular trafficking of cellular organelles in cultured cell lines suggested that tau inhibits AT (Stamer et al., 2002). *In vitro* studies using purified components of the AT system further supported a model where tau would elicit this effect by competing with conventional kinesin heavy chain subunits for microtubule binding (Seitz et al., 2002; Mandelkow et al., 2003). However, studies in the squid axoplasm preparation reveal that even supraphysiological (i.e., ~20-fold higher) levels of human tau monomers do not negatively impact AT (Morfini et al., 2007). Pulse chase studies *in vivo* extend the conclusions from squid axoplasm studies to mammalian neurons, showing normal AT rates in the optic nerve of tau-overexpressing mice (Yuan et al., 2008).

The highly dynamic nature of the interaction between tau and microtubules *in vitro* and *in situ* also appears inconsistent with the notion that tau physically blocks motor proteins (Samsonov et al., 2004; Janning et al., 2014; Stern et al., 2017). Recent work suggests that disease-related phosphorylation does not cause tau to fall off microtubules as the off-rate is unaffected by pseudophosphorylation at the PHF1 site (i.e., S396 and S404), but may reduce microtubule binding by decreasing the on-rate (Niewidok et al., 2016). The highly dynamic conformational flexibility of tau likely facilitates its interaction with a large repertoire of binding partners (Jeganathan et al., 2006; Uversky, 2015; Stern et al., 2017) (reviewed in Bakota et al., 2017). Further, tau's role as a scaffolding protein and the recruitment of phosphotransferases to the microtubule cystoskeleton associated with this function could in turn be subject to phosphoregulation.

The major motor proteins responsible for AT, conventional kinesin and cytoplasmic dynein, are regulated by specific protein kinases providing a novel mechanism linking pathological forms of tau to deficits in AT (Kanaan et al., 2013; Gibbs et al., 2015; Brady and Morfini, 2017). Unlike soluble tau monomers, tau aggregates inhibit anterograde AT in the isolated squid axoplasm when perfused at physiological levels (LaPointe et al., 2009). Remarkably, this effect is dependent upon amino acids 2–18 in the extreme amino terminus of tau, a motif termed the phosphatase-activating domain (PAD) (Kanaan et al., 2011). PAD is sufficient to activate protein phosphatase 1 (PP1) and GSK3 in a manner independent of microtubule binding (Kanaan et al., 2011). PP1 reportedly activates GSK3β by dephosphorylating the autoinhibitory residue (Ser 9), causing its activation (Morfini et al., 2004). Active GSK3 in turn phosphorylates kinesin light chain subunits of conventional kinesin, promoting release of transported cargoes from this motor protein (Morfini et al., 2002b). Systematic evaluation of the effects that numerous tau construct variants elicit on AT reveals that different pathological changes (e.g., mutations, aggregation, oligomerization and phosphorylation) promote increased PAD exposure (Figure 1A; Kanaan et al., 2012). Given the unique dependence of axons on sustained AT, these studies highlight a specific kinase-dependent mechanism by which pathological forms of tau may cause axonal dysfunction, further supporting a role of tau as a modulator of protein kinases involved in AT regulation (Figure 1B). More recent studies provide further support for this mechanism in mammalian neurons by showing that amyloid-β oligomer-mediated impairments in AT require both tau and specific phosphotransferases (Decker et al., 2010; Vossel et al., 2015). Additionally, phosphorylation of tau at specific epitopes modulates both its dynamic behavior and its effect on AT (Kanaan et al., 2012; Tiernan et al., 2016; Stern et al., 2017). For example, tyrosine 18 phosphorylation within PAD by non-receptor kinases blocks the inhibitory effect of pathogenic tau on anterograde AT (Kanaan et al., 2012). In contrast, phospho-mimicking residues within the proline-rich region and the C-terminus confer a toxic effect on anterograde AT upon wild type tau monomers (Kanaan et al., 2011; Tiernan et al., 2016; Stern et al., 2017). Interestingly, tau-induced toxicity in cultured cortical astrocytes is associated with kinesin-dependent transport deficits (Yoshiyama et al., 2003), suggesting that tau may impair microtubule-dependent transport in glial cells as well.

In addition to the mechanisms above, mechanisms linking pathological tau to Ca^2+^ dysregulation may negatively impact axonal connectivity (Figure 1F). For example, injection of wild-type tau in the squid giant synapse promotes release of Ca^2+^ from the endoplasmic reticulum, leading to inhibition of synaptic vesicle exocytosis and synaptic transmission through a mechanism involving PAD exposure and GSK3 activation (Figure 1F; Moreno et al., 2016). Oligomeric tau is particularly toxic to synaptic function, causing impaired long-term potentiation (Fa et al., 2016) and long-term depression (Decker et al., 2015) in cultured neurons and mouse brain slice cultures, as well as memory deficits in living mice (Fa et al., 2016). Studies also show that tau oligomers can cause Ca^2+^ imbalance and cell death in induced pluripotent stem cells (Imamura et al., 2016), and tau may further increase Ca^2+^ levels by directly inhibiting plasma membrane Ca^2+^ ATPase (Berrocal et al., 2016). Besides the endoplasmic reticulum, mitochondria are a main source of Ca^2+^ buffering in cells (Werth and Thayer, 1994). Interestingly, P301L mutant tau reduces the number of axonal mitochondria (Rodriguez-Martin et al., 2016) and the N-terminal domain of tau may stimulate autophagic turnover of mitochondria in neurons (Amadoro et al., 2014). Reduced numbers and/or dysfunction of axonal mitochondria could in turn promote abnormal Ca^2+^ levels (reviewed in Eckert et al., 2014). Ultimately, pathogenic tau-mediated increases in Ca^2+^ levels may promote abnormal activation of calcium-activated proteases and proteolysis of critical cytoskeletal protein components (Figure 1F; Yin et al., 2016). Consistent with this notion, increased calpain activation occurs in AD and other tauopathy brains (Adamec et al., 2002), and inhibition of calcium activated protease reduces axonal degeneration in the P301L transgenic mouse model (Rao et al., 2014). Interestingly, studies suggest that microtubules devoid of tau are more vulnerable to degradation by the microtubule-severing protein katanin, providing another route by which abnormal tau modifications promote cytoskeletal degradation (Figure 1E; Qiang et al., 2006; Sudo and Baas, 2011). Collectively, these studies suggest various mechanism linking pathogenic forms of tau to axonal degeneration. A better understanding of these mechanisms may provide a broader set of tau-based therapeutic targets.

## Conclusions

The landscape of tauopathies is marked by significant heterogeneity in clinical presentation, cellular topography, and neuropathological features. Despite this diversity, an examinations of human brains and animal models of tauopathies reveal axonopathy as a common feature of these diverse diseases (Hyman et al., 1990; Ahmed et al., 2008; Kovacs et al., 2008; Ling et al., 2015). A comprehensive analysis demonstrates that tau likely plays a central role in axonal dysfunction and degeneration and that disease-related modifications of tau contribute to axonopathy and synaptic dysfunction through multiple pathways including misregulation of phosphotransferase activities, AT deficits, disruption of Ca^2+^ homeostasis, altered glial function, and others (Figure 1). Developing and implementing therapeutic strategies based on preserving neuronal connectivity will require a continued deepening of our understanding of these mechanisms and the identification of specific molecular components linking pathological tau to axonopathy in the context of each tauopathy.

## Author contributions

AK, BC, KC, GM, and NK all contributed equally with writing, editing and figure preparation for the manuscript.

### Conflict of interest statement

The authors declare that the research was conducted in the absence of any commercial or financial relationships that could be construed as a potential conflict of interest.

## Abbreviations

PET: Positron emission tomography
DTI: Diffuse tensor imaging
MRI: Magnetic resonance imaging
AD: Alzheimer's disease
FTDP-17: Frontal temporal dementia with parkinsonism linked to chromosome 17
CTE: Chronic traumatic encephalopathy
PSP: Progressive supranuclear palsy
CBD: Corticobasal degeneration
PiD: Pick's disease.

## References

[B1] Adamec E. Mohan P. Vonsattel J. P. Nixon R. A. (2002). Calpain activation in neurodegenerative diseases: confocal immunofluorescence study with antibodies specifically recognizing the active form of calpain 2. Acta Neuropathol. 104, 92–104. 10.1007/s00401-002-0528-6 12070670

[B2] Ahmed Z. Josephs K. A. Gonzalez J. Delledonne A. Dickson D. W. (2008). Clinical and neuropathologic features of progressive supranuclear palsy with severe pallido-nigro-luysial degeneration and axonal dystrophy. Brain 131, 460–472. 10.1093/brain/awm301 18158316

[B3] Allen B. Ingram E. Takao M. Smith M. J. Jakes R. Virdee K.. (2002). Abundant tau filaments and nonapoptotic neurodegeneration in transgenic mice expressing human P301S tau protein. J. Neurosci. 22, 9340–9351. 12417659 10.1523/JNEUROSCI.22-21-09340.2002 PMC6758022

[B4] Amadoro G. Corsetti V. Florenzano F. Atlante A. Ciotti M. T. Mongiardi M. P.. (2014). AD-linked, toxic NH2 human tau affects the quality control of mitochondria in neurons. Neurobiol. Dis. 62, 489–507. 10.1016/j.nbd.2013.10.018 24411077

[B5] Andorfer C. Kress Y. Espinoza M. De Silva R. Tucker K. L. Barde Y. A.. (2003). Hyperphosphorylation and aggregation of tau in mice expressing normal human tau isoforms. J. Neurochem. 86, 582–590. 10.1046/j.1471-4159.2003.01879.x 12859672

[B6] Arendt T. Stieler J. T. Holzer M. (2016). Tau and tauopathies. Brain Res. Bull. 126, 238–292. 10.1016/j.brainresbull.2016.08.018 27615390

[B7] Baas P. W. Rao A. N. Matamoros A. J. Leo L. (2016). Stability properties of neuronal microtubules. Cytoskeleton (Hoboken) 73, 442–460. 10.1002/cm.21286 26887570 PMC5541393

[B8] Bakota L. Ussif A. Jeserich G. Brandt R. (2017). Systemic and network functions of the microtubule-associated protein tau: implications for tau-based therapies. Mol. Cell. Neurosci. 16, 30257–30263. 10.1016/j.mcn.2017.03.003 28318914

[B9] Ballatore C. Lee V. M. Trojanowski J. Q. (2007). Tau-mediated neurodegeneration in Alzheimer's disease and related disorders. Nat. Rev. Neurosci. 8, 663–672. 10.1038/nrn2194 17684513

[B10] Barres B. A. Jacobson M. D. Schmid R. Sendtner M. Raff M. C. (1993). Does oligodendrocyte survival depend on axons? Curr. Biol. 3, 489–497. 10.1016/0960-9822(93)90039-Q 15335686

[B11] Barrio J. R. Small G. W. Wong K. P. Huang S. C. Liu J. Merrill D. A. (2015). *In vivo* characterization of chronic traumatic encephalopathy using [F-18]FDDNP PET brain imaging. Proc. Natl. Acad. Sci. U.S.A. 112, E2039–E2047. 10.1073/pnas.1409952112 25848027 PMC4413350

[B12] Berrocal M. Corbacho I. Sepulveda M. R. Gutierrez-Merino C. Mata A. M. (2016). Phospholipids and calmodulin modulate the inhibition of PMCA activity by tau. Biochim. Biophys. Acta 1864, 1028–1035. 10.1016/j.bbamcr.2016.10.023 27818274

[B13] Black M. M. (2016). Axonal transport: the orderly motion of axonal structures. Methods Cell Biol. 131, 1–19. 10.1016/bs.mcb.2015.06.001 26794507

[B14] Blennow K. Brody D. L. Kochanek P. M. Levin H. Mckee A. Ribbers G. M.. (2016). Traumatic brain injuries. Nat. Rev. Dis. Primers 2:16084. 10.1038/nrdp.2016.84 27853132

[B15] Blumbergs P. C. Scott G. Manavis J. Wainwright H. Simpson D. A. Mclean A. J. (1994). Staining of amyloid precursor protein to study axonal damage in mild head injury. Lancet 344, 1055–1056. 10.1016/S0140-6736(94)91712-4 7523810

[B16] Borroni B. Garibotto V. Agosti C. Brambati S. M. Bellelli G. Gasparotti R.. (2008). White matter changes in corticobasal degeneration syndrome and correlation with limb apraxia. Arch. Neurol. 65, 796–801. 10.1001/archneur.65.6.796 18541800

[B17] Bozzali M. Falini A. Franceschi M. Cercignani M. Zuffi M. Scotti G.. (2002). White matter damage in Alzheimer's disease assessed *in vivo* using diffusion tensor magnetic resonance imaging. J. Neurol. Neurosurg. Psychiatr. 72, 742–746. 10.1136/jnnp.72.6.742 12023417 PMC1737921

[B18] Braak E. Arai K. Braak H. (1999). Cerebellar involvement in Pick's disease: affliction of mossy fibers, monodendritic brush cells, and dentate projection neurons. Exp. Neurol. 159, 153–163. 10.1006/exnr.1999.7131 10486184

[B19] Brady S. T. Morfini G. A. (2017). Regulation of motor proteins, axonal transport deficits and adult-onset neurodegenerative diseases. Neurobiol. Dis. 105, 273–282. 10.1016/j.nbd.2017.04.010 28411118 PMC5522763

[B20] Brandt R. Leger J. Lee G. (1995). Interaction of tau with the neural plasma membrane mediated by tau's amino-terminal projection domain. J. Cell Biol. 131, 1327–1340. 10.1083/jcb.131.5.1327 8522593 PMC2120645

[B21] Brion J. P. Tremp G. Octave J. N. (1999). Transgenic expression of the shortest human tau affects its compartmentalization and its phosphorylation as in the pretangle stage of Alzheimer's disease. Am. J. Pathol. 154, 255–270. 10.1016/S0002-9440(10)65272-8 9916940 PMC1853433

[B22] Caillierez R. Begard S. Lecolle K. Deramecourt V. Zommer N. Dujardin S.. (2013). Lentiviral delivery of the human wild-type tau protein mediates a slow and progressive neurodegenerative tau pathology in the rat brain. Mol. Ther. 21, 1358–1368. 10.1038/mt.2013.66 23609018 PMC3702115

[B23] Carmel G. Mager E. M. Binder L. I. Kuret J. (1996). The structural basis of monoclonal antibody Alz50's selectivity for Alzheimer's disease pathology. J. Biol. Chem. 271, 32789–32795. 10.1074/jbc.271.51.32789 8955115

[B24] Cheng H. C. Ulane C. M. Burke R. E. (2010). Clinical progression in Parkinson disease and the neurobiology of axons. Ann. Neurol. 67, 715–725. 10.1002/ana.21995 20517933 PMC2918373

[B25] Chin S. S. Goldman J. E. (1996). Glial inclusions in CNS degenerative diseases. J. Neuropathol. Exp. Neurol. 55, 499–508. 10.1097/00005072-199605000-00001 8627339

[B26] Cleveland D. W. Hwo S. Y. Kirschner M. W. (1977a). Physical and chemical properties of purified tau factor and the role of tau in microtubule assembly. J. Mol. Biol. 116, 227–247. 10.1016/0022-2836(77)90214-5 146092

[B27] Cleveland D. W. Hwo S. Y. Kirschner M. W. (1977b). Purification of tau, a microtubule-associated protein that induces assembly of microtubules from purified tubulin. J. Mol. Biol. 116, 207–225. 10.1016/0022-2836(77)90213-3 599557

[B28] Cochran E. J. Fox J. H. Mufson E. J. (1994). Severe panencephalic Pick's disease with Alzheimer's disease-like neuropil threads and synaptophysin immunoreactivity. Acta Neuropathol. 88, 479–484. 10.1007/BF00389503 7847079

[B29] Combs B. Hamel C. Kanaan N. M. (2016a). Pathological conformations involving the amino terminus of tau occur early in Alzheimer's disease and are differentially detected by monoclonal antibodies. Neurobiol. Dis. 94, 18–31. 10.1016/j.nbd.2016.05.016 27260838 PMC4983528

[B30] Combs B. Kneynsberg A. Kanaan N. M. (2016b). Gene therapy models of Alzheimer's disease and other dementias. Methods Mol. Biol. 1382, 339–366. 10.1007/978-1-4939-3271-9_25 26611599 PMC4734109

[B31] Conde C. Caceres A. (2009). Microtubule assembly, organization and dynamics in axons and dendrites. Nat. Rev. Neurosci. 10, 319–332. 10.1038/nrn2631 19377501

[B32] Cox K. Combs B. Abdelmesih B. Morfini G. Brady S. T. Kanaan N. M. (2016). Analysis of isoform-specific tau aggregates suggests a common toxic mechanism involving similar pathological conformations and axonal transport inhibition. Neurobiol. Aging 47, 113–126. 10.1016/j.neurobiolaging.2016.07.015 27574109 PMC5075521

[B33] Dawson H. N. Ferreira A. Eyster M. V. Ghoshal N. Binder L. I. Vitek M. P. (2001). Inhibition of neuronal maturation in primary hippocampal neurons from tau deficient mice. J. Cell Sci. 114, 1179–1187. 11228161 10.1242/jcs.114.6.1179

[B34] de Calignon A. Polydoro M. Suarez-Calvet M. William C. Adamowicz D. H. Kopeikina K. J.. (2012). Propagation of tau pathology in a model of early Alzheimer's disease. Neuron 73, 685–697. 10.1016/j.neuron.2011.11.033 22365544 PMC3292759

[B35] Decker H. Lo K. Y. Unger S. M. Ferreira S. T. Silverman M. A. (2010). Amyloid-beta peptide oligomers disrupt axonal transport through an NMDA receptor-dependent mechanism that is mediated by glycogen synthase kinase 3beta in primary cultured hippocampal neurons. J. Neurosci. 30, 9166–9171. 10.1523/JNEUROSCI.1074-10.2010 20610750 PMC6632489

[B36] Decker J. M. Kruger L. Sydow A. Zhao S. Frotscher M. Mandelkow E.. (2015). Pro-aggregant Tau impairs mossy fiber plasticity due to structural changes and Ca(++) dysregulation. Acta Neuropathol. Commun. 3, 23. 10.1186/s40478-015-0193-3 25853683 PMC4384391

[B37] Dekosky S. T. Scheff S. W. (1990). Synapse loss in frontal cortex biopsies in Alzheimer's disease: correlation with cognitive severity. Ann. Neurol. 27, 457–464. 10.1002/ana.410270502 2360787

[B38] Delisle M. B. Murrell J. R. Richardson R. Trofatter J. A. Rascol O. Soulages X.. (1999). A mutation at codon 279 (N279K) in exon 10 of the Tau gene causes a tauopathy with dementia and supranuclear palsy. Acta Neuropathol. 98, 62–77. 10.1007/s004010051052 10412802

[B39] Dessi F. Colle M. A. Hauw J. J. Duyckaerts C. (1997). Accumulation of SNAP-25 immunoreactive material in axons of Alzheimer's disease. Neuroreport 8, 3685–3689. 10.1097/00001756-199712010-00006 9427351

[B40] Dickson D. W. (1998). Pick's disease: a modern approach. Brain Pathol. 8, 339–354. 10.1111/j.1750-3639.1998.tb00158.x 9546291 PMC8098155

[B41] Dickson D. W. (1999). Neuropathologic differentiation of progressive supranuclear palsy and corticobasal degeneration. J. Neurol. 246(Suppl. 2), II6–II15. 10.1007/BF03161076 10525997

[B42] Dickson D. W. Feany M. B. Yen S. H. Mattiace L. A. Davies P. (1996). Cytoskeletal pathology in non-Alzheimer degenerative dementia: new lesions in diffuse Lewy body disease, Pick's disease, and corticobasal degeneration. J. Neural Transm. Suppl. 47, 31–46. 10.1007/978-3-7091-6892-9_2 8841955

[B43] Doi T. Iwasa K. Makifuchi T. Takamori M. (1999). White matter hyperintensities on MRI in a patient with corticobasal degeneration. Acta Neurol. Scand. 99, 199–201. 10.1111/j.1600-0404.1999.tb07345.x 10100966

[B44] Dopper E. G. Rombouts S. A. Jiskoot L. C. Den Heijer T. De Graaf J. R. De Koning I.. (2014). Structural and functional brain connectivity in presymptomatic familial frontotemporal dementia. Neurology 83, e19–e26. 10.1212/WNL.0000000000000583 25002573

[B45] Duff K. Knight H. Refolo L. M. Sanders S. Yu X. Picciano M.. (2000). Characterization of pathology in transgenic mice over-expressing human genomic and cDNA tau transgenes. Neurobiol. Dis. 7, 87–98. 10.1006/nbdi.1999.0279 10783293

[B46] Dumanchin C. Camuzat A. Campion D. Verpillat P. Hannequin D. Dubois B.. (1998). Segregation of a missense mutation in the microtubule-associated protein tau gene with familial frontotemporal dementia and parkinsonism. Hum. Mol. Genet. 7, 1825–1829. 10.1093/hmg/7.11.1825 9736786

[B47] Eckert A. Nisbet R. Grimm A. Gotz J. (2014). March separate, strike together–role of phosphorylated TAU in mitochondrial dysfunction in Alzheimer's disease. Biochim. Biophys. Acta 1842, 1258–1266. 10.1016/j.bbadis.2013.08.013 24051203

[B48] Fa M. Puzzo D. Piacentini R. Staniszewski A. Zhang H. Baltrons M. A.. (2016). Extracellular Tau oligomers produce an immediate impairment of LTP and memory. Sci. Rep. 6:19393. 10.1038/srep19393 26786552 PMC4726138

[B49] Feany M. B. Dickson D. W. (1995). Widespread cytoskeletal pathology characterizes corticobasal degeneration. Am. J. Pathol. 146, 1388–1396. 7778678 PMC1870913

[B50] Feinstein S. C. Wilson L. (2005). Inability of tau to properly regulate neuronal microtubule dynamics: a loss-of-function mechanism by which tau might mediate neuronal cell death. Biochim. Biophys. Acta 1739, 268–279. 10.1016/j.bbadis.2004.07.002 15615645

[B51] Foster N. L. Wilhelmsen K. Sima A. A. Jones M. Z. D'amato C. J. Gilman S. (1997). Frontotemporal dementia and parkinsonism linked to chromosome 17: a consensus conference. Conference participants. Ann. Neurol 41, 706–715. 10.1002/ana.410410606 9189031

[B52] Fujio J. Hosono H. Ishiguro K. Ikegami S. Fujita S. C. (2007). Tau phosphorylation in the mouse brain during aversive conditioning. Neurochem. Int. 51, 200–208. 10.1016/j.neuint.2007.04.024 17597257

[B53] Gatto R. G. Chu Y. Ye A. Q. Price S. D. Tavassoli E. Buenaventura A.. (2015). Analysis of YFP(J16)-R6/2 reporter mice and postmortem brains reveals early pathology and increased vulnerability of callosal axons in Huntington's disease. Hum. Mol. Genet. 24, 5285–5298. 10.1093/hmg/ddv248 26123489 PMC4550824

[B54] Ghetti B. Oblak A. L. Boeve B. F. Johnson K. A. Dickerson B. C. Goedert M. (2015). Invited review: frontotemporal dementia caused by microtubule-associated protein tau gene (MAPT) mutations: a chameleon for neuropathology and neuroimaging. Neuropathol. Appl. Neurobiol. 41, 24–46. 10.1111/nan.12213 25556536 PMC4329416

[B55] Ghoshal N. Garcia-Sierra F. Wuu J. Leurgans S. Bennett D. A. Berry R. W.. (2002). Tau conformational changes correspond to impairments of episodic memory in mild cognitive impairment and Alzheimer's disease. Exp. Neurol. 177, 475–493. 10.1006/exnr.2002.8014 12429193

[B56] Gibbs K. L. Greensmith L. Schiavo G. (2015). Regulation of axonal transport by protein kinases. Trends Biochem. Sci. 40, 597–610. 10.1016/j.tibs.2015.08.003 26410600

[B57] Giza C. C. Hovda D. A. (2001). The Neurometabolic Cascade of Concussion. J. Athl. Train. 36, 228–235. 12937489 PMC155411

[B58] Gotz J. Chen F. Van Dorpe J. Nitsch R. M. (2001). Formation of neurofibrillary tangles in P301l tau transgenic mice induced by Abeta 42 fibrils. Science 293, 1491–1495. 10.1126/science.1062097 11520988

[B59] Gotz J. Deters N. Doldissen A. Bokhari L. Ke Y. Wiesner A.. (2007). A decade of tau transgenic animal models and beyond. Brain Pathol. 17, 91–103. 10.1111/j.1750-3639.2007.00051.x 17493043 PMC8095624

[B60] Gotz J. Ittner L. M. Kins S. (2006). Do axonal defects in tau and amyloid precursor protein transgenic animals model axonopathy in Alzheimer's disease? J. Neurochem. 98, 993–1006. 10.1111/j.1471-4159.2006.03955.x 16787410

[B61] Gotz J. Probst A. Spillantini M. G. Schafer T. Jakes R. Burki K.. (1995). Somatodendritic localization and hyperphosphorylation of tau protein in transgenic mice expressing the longest human brain tau isoform. EMBO J. 14, 1304–1313. 7729409 10.1002/j.1460-2075.1995.tb07116.x PMC398215

[B62] Guo T. Noble W. Hanger D. P. (2017). Roles of tau protein in health and disease. Acta Neuropathol. 133, 665–704. 10.1007/s00401-017-1707-9 28386764 PMC5390006

[B63] Hampel H. Teipel S. J. Alexander G. E. Horwitz B. Teichberg D. Schapiro M. B.. (1998). Corpus callosum atrophy is a possible indicator of region- and cell type-specific neuronal degeneration in Alzheimer disease: a magnetic resonance imaging analysis. Arch. Neurol. 55, 193–198. 10.1001/archneur.55.2.193 9482361

[B64] Harada A. Oguchi K. Okabe S. Kuno J. Terada S. Ohshima T.. (1994). Altered microtubule organization in small-calibre axons of mice lacking tau protein. Nature 369, 488–491. 10.1038/369488a0 8202139

[B65] Hauw J. J. Daniel S. E. Dickson D. Horoupian D. S. Jellinger K. Lantos P. L.. (1994). Preliminary NINDS neuropathologic criteria for Steele-Richardson-Olszewski syndrome (progressive supranuclear palsy). Neurology 44, 2015–2019. 10.1212/WNL.44.11.2015 7969952

[B66] Hauw J. J. Verny M. Delaere P. Cervera P. He Y. Duyckaerts C. (1990). Constant neurofibrillary changes in the neocortex in progressive supranuclear palsy. Basic differences with Alzheimer's disease and aging. Neurosci. Lett. 119, 182–186. 10.1016/0304-3940(90)90829-X 1704110

[B67] Hebron M. L. Algarzae N. K. Lonskaya I. Moussa C. (2014). Fractalkine signaling and Tau hyper-phosphorylation are associated with autophagic alterations in lentiviral Tau and Abeta1-42 gene transfer models. Exp. Neurol. 251, 127–138. 10.1016/j.expneurol.2013.01.009 23333589 PMC3644355

[B68] Higuchi M. Lee V. M. Trojanowski J. Q. (2002). Tau and axonopathy in neurodegenerative disorders. Neuromolecular Med. 2, 131–150. 10.1385/NMM:2:2:131 12428808

[B69] Higuchi M. Zhang B. Forman M. S. Yoshiyama Y. Trojanowski J. Q. Lee V. M. (2005). Axonal degeneration induced by targeted expression of mutant human tau in oligodendrocytes of transgenic mice that model glial tauopathies. J. Neurosci. 25, 9434–9443. 10.1523/JNEUROSCI.2691-05.2005 16221853 PMC6725712

[B70] Huang J. Auchus A. P. (2007). Diffusion tensor imaging of normal appearing white matter and its correlation with cognitive functioning in mild cognitive impairment and Alzheimer's disease. Ann. N. Y. Acad. Sci. 1097, 259–264. 10.1196/annals.1379.021 17413027

[B71] Hutton M. Lendon C. L. Rizzu P. Baker M. Froelich S. Houlden H.. (1998). Association of missense and 5′-splice-site mutations in tau with the inherited dementia FTDP-17. Nature 393, 702–705. 10.1038/31508 9641683

[B72] Hyman B. T. Van Hoesen G. W. Damasio A. R. (1990). Memory-related neural systems in Alzheimer's disease: an anatomic study. Neurology 40, 1721–1730. 10.1212/WNL.40.11.1721 2234428

[B73] Ihara M. Polvikoski T. M. Hall R. Slade J. Y. Perry R. H. Oakley A. E.. (2010). Quantification of myelin loss in frontal lobe white matter in vascular dementia, Alzheimer's disease, and dementia with Lewy bodies. Acta Neuropathol. 119, 579–589. 10.1007/s00401-009-0635-8 20091409 PMC2849937

[B74] Ikeda K. Akiyama H. Haga C. Kondo H. Arima K. Oda T. (1994). Argyrophilic thread-like structure in corticobasal degeneration and supranuclear palsy. Neurosci. Lett. 174, 157–159. 10.1016/0304-3940(94)90010-8 7526285

[B75] Ikegami S. Harada A. Hirokawa N. (2000). Muscle weakness, hyperactivity, and impairment in fear conditioning in tau-deficient mice. Neurosci. Lett. 279, 129–132. 10.1016/S0304-3940(99)00964-7 10688046

[B76] Imamura K. Sahara N. Kanaan N. M. Tsukita K. Kondo T. Kutoku Y.. (2016). Calcium dysregulation contributes to neurodegeneration in FTLD patient iPSC-derived neurons. Sci. Rep. 6:34904. 10.1038/srep34904 27721502 PMC5056519

[B77] Ishihara T. Hong M. Zhang B. Nakagawa Y. Lee M. K. Trojanowski J. Q.. (1999). Age-dependent emergence and progression of a tauopathy in transgenic mice overexpressing the shortest human tau isoform. Neuron 24, 751–762. 10.1016/S0896-6273(00)81127-7 10595524

[B78] Ishizawa K. Lin W. L. Tiseo P. Honer W. G. Davies P. Dickson D. W. (2000). A qualitative and quantitative study of grumose degeneration in progressive supranuclear palsy. J. Neuropathol. Exp. Neurol. 59, 513–524. 10.1093/jnen/59.6.513 10850864

[B79] Ittner L. M. Ke Y. D. Delerue F. Bi M. Gladbach A. Van Eersel J.. (2010). Dendritic function of tau mediates amyloid-beta toxicity in Alzheimer's disease mouse models. Cell 142, 387–397. 10.1016/j.cell.2010.06.036 20655099

[B80] Janning D. Igaev M. Sundermann F. Bruhmann J. Beutel O. Heinisch J. J.. (2014). Single-molecule tracking of tau reveals fast kiss-and-hop interaction with microtubules in living neurons. Mol. Biol. Cell 25, 3541–3551. 10.1091/mbc.E14-06-1099 25165145 PMC4230615

[B81] Jaworski T. Dewachter I. Lechat B. Croes S. Termont A. Demedts D.. (2009). AAV-tau mediates pyramidal neurodegeneration by cell-cycle re-entry without neurofibrillary tangle formation in wild-type mice. PLoS ONE 4:e7280. 10.1371/journal.pone.0007280 19794916 PMC2748684

[B82] Jaworski T. Lechat B. Demedts D. Gielis L. Devijver H. Borghgraef P.. (2011). Dendritic degeneration, neurovascular defects, and inflammation precede neuronal loss in a mouse model for tau-mediated neurodegeneration. Am. J. Pathol. 179, 2001–2015. 10.1016/j.ajpath.2011.06.025 21839061 PMC3181369

[B83] Jeganathan S. Hascher A. Chinnathambi S. Biernat J. Mandelkow E. M. Mandelkow E. (2008). Proline-directed pseudo-phosphorylation at AT8 and PHF1 epitopes induces a compaction of the paperclip folding of Tau and generates a pathological (MC-1) conformation. J. Biol. Chem. 283, 32066–32076. 10.1074/jbc.M805300200 18725412

[B84] Jeganathan S. Von Bergen M. Brutlach H. Steinhoff H. J. Mandelkow E. (2006). Global hairpin folding of tau in solution. Biochemistry 45, 2283–2293. 10.1021/bi0521543 16475817

[B85] Kahlson M. A. Colodner K. J. (2015). Glial Tau pathology in tauopathies: functional consequences. J. Exp. Neurosci. 9, 43–50. 10.4137/JEN.S25515 26884683 PMC4750898

[B86] Kanaan N. M. Cox K. Alvarez V. E. Stein T. D. Poncil S. Mckee A. C. (2016). Characterization of early pathological tau conformations and phosphorylation in chronic traumatic encephalopathy. J. Neuropathol. Exp. Neurol. 75, 19–34. 10.1093/jnen/nlv001 26671985 PMC4891281

[B87] Kanaan N. M. Morfini G. A. Lapointe N. E. Pigino G. F. Patterson K. R. Song Y.. (2011). Pathogenic forms of tau inhibit kinesin-dependent axonal transport through a mechanism involving activation of axonal phosphotransferases. J. Neurosci. 31, 9858–9868. 10.1523/JNEUROSCI.0560-11.2011 21734277 PMC3391724

[B88] Kanaan N. M. Morfini G. Pigino G. Lapointe N. E. Andreadis A. Song Y.. (2012). Phosphorylation in the amino terminus of tau prevents inhibition of anterograde axonal transport. Neurobiol. Aging 33, 826 e815–830. 10.1016/j.neurobiolaging.2011.06.006 21794954 PMC3272324

[B89] Kanaan N. M. Pigino G. F. Brady S. T. Lazarov O. Binder L. I. Morfini G. A. (2013). Axonal degeneration in Alzheimer's disease: when signaling abnormalities meet the axonal transport system. Exp. Neurol. 246, 44–53. 10.1016/j.expneurol.2012.06.003 22721767 PMC3465504

[B90] Ke Y. D. Suchowerska A. K. Van Der Hoven J. De Silva D. M. Wu C. W. Van Eersel J.. (2012). Lessons from tau-deficient mice. Int. J. Alzheimers. Dis. 2012:873270. 10.1155/2012/873270 22720190 PMC3375147

[B91] Kiernan P. T. Montenigro P. H. Solomon T. M. Mckee A. C. (2015). Chronic traumatic encephalopathy: a neurodegenerative consequence of repetitive traumatic brain injury. Semin. Neurol. 35, 20–28. 10.1055/s-0035-1545080 25714864

[B92] Knake S. Belke M. Menzler K. Pilatus U. Eggert K. M. Oertel W. H.. (2010). *In vivo* demonstration of microstructural brain pathology in progressive supranuclear palsy: a DTI study using TBSS. Mov. Disord. 25, 1232–1238. 10.1002/mds.23054 20222139

[B93] Kneynsberg A. Collier T. J. Manfredsson F. P. Kanaan N. M. (2016). Quantitative and semi-quantitative measurements of axonal degeneration in tissue and primary neuron cultures. J. Neurosci. Methods 266, 32–41. 10.1016/j.jneumeth.2016.03.004 27031947 PMC4874894

[B94] Koerte I. K. Ertl-Wagner B. Reiser M. Zafonte R. Shenton M. E. (2012a). White matter integrity in the brains of professional soccer players without a symptomatic concussion. JAMA 308, 1859–1861. 10.1001/jama.2012.13735 23150002 PMC4103415

[B95] Koerte I. K. Kaufmann D. Hartl E. Bouix S. Pasternak O. Kubicki M. (2012b). A prospective study of physician-observed concussion during a varsity university hockey season: white matter integrity in ice hockey players. Part 3 of 4. Neurosurg. Focus 33, E3:1–7. 10.3171/2012.10.FOCUS12303 PMC5687247 23199426

[B96] Kouri N. Carlomagno Y. Baker M. Liesinger A. M. Caselli R. J. Wszolek Z. K.. (2014). Novel mutation in MAPT exon 13 (p.N410H) causes corticobasal degeneration. Acta Neuropathol. 127, 271–282. 10.1007/s00401-013-1193-7 24121548 PMC3943649

[B97] Kouri N. Whitwell J. L. Josephs K. A. Rademakers R. Dickson D. W. (2011). Corticobasal degeneration: a pathologically distinct 4R tauopathy. Nat. Rev. Neurol. 7, 263–272. 10.1038/nrneurol.2011.43 21487420 PMC10006729

[B98] Kovacs G. G. Majtenyi K. Spina S. Murrell J. R. Gelpi E. Hoftberger R.. (2008). White matter tauopathy with globular glial inclusions: a distinct sporadic frontotemporal lobar degeneration. J. Neuropathol. Exp. Neurol. 67, 963–975. 10.1097/NEN.0b013e318187a80f 18800011 PMC2785030

[B99] Kowall N. W. Kosik K. S. (1987). Axonal disruption and aberrant localization of tau protein characterize the neuropil pathology of Alzheimer's disease. Ann. Neurol. 22, 639–643. 10.1002/ana.410220514 3122646

[B100] Kraus M. F. Susmaras T. Caughlin B. P. Walker C. J. Sweeney J. A. Little D. M. (2007). White matter integrity and cognition in chronic traumatic brain injury: a diffusion tensor imaging study. Brain 130, 2508–2519. 10.1093/brain/awm216 17872928

[B101] LaFerla F. M. Green K. N. (2012). Animal models of Alzheimer disease. Cold Spring Harb. Perspect. Med. 2:a006320. 10.1101/cshperspect.a006320 23002015 PMC3543097

[B102] LaPointe N. E. Morfini G. Pigino G. Gaisina I. N. Kozikowski A. P. Binder L. I.. (2009). The amino terminus of tau inhibits kinesin-dependent axonal transport: implications for filament toxicity. J. Neurosci. Res. 87, 440–451. 10.1002/jnr.21850 18798283 PMC2739042

[B103] Lau D. H. Hogseth M. Phillips E. C. O'neill M. J. Pooler A. M. Noble W.. (2016). Critical residues involved in tau binding to fyn: implications for tau phosphorylation in Alzheimer's disease. Acta Neuropathol. Commun. 4, 49. 10.1186/s40478-016-0317-4 27193083 PMC4870772

[B104] Lee G. Newman S. T. Gard D. L. Band H. Panchamoorthy G. (1998). Tau interacts with src-family non-receptor tyrosine kinases. J Cell Sci. 111(Pt 21), 3167–3177. 9763511 10.1242/jcs.111.21.3167

[B105] Lei P. Ayton S. Moon S. Zhang Q. Volitakis I. Finkelstein D. I.. (2014). Motor and cognitive deficits in aged tau knockout mice in two background strains. Mol. Neurodegener. 9:29. 10.1186/1750-1326-9-29 25124182 PMC4141346

[B106] Lewis J. Mcgowan E. Rockwood J. Melrose H. Nacharaju P. Van Slegtenhorst M.. (2000). Neurofibrillary tangles, amyotrophy and progressive motor disturbance in mice expressing mutant (P301L) tau protein. Nat. Genet. 25, 402–405. 10.1038/78078 10932182

[B107] Li T. Hawkes C. Qureshi H. Y. Kar S. Paudel H. K. (2006). Cyclin-dependent protein kinase 5 primes microtubule-associated protein tau site-specifically for glycogen synthase kinase 3beta. Biochemistry 45, 3134–3145. 10.1021/bi051635j 16519508

[B108] Liao H. Li Y. Brautigan D. L. Gundersen G. G. (1998). Protein phosphatase 1 is targeted to microtubules by the microtubule-associated protein Tau. J. Biol. Chem. 273, 21901–21908. 10.1074/jbc.273.34.21901 9705329

[B109] Lin W. L. Lewis J. Yen S. H. Hutton M. Dickson D. W. (2003). Ultrastructural neuronal pathology in transgenic mice expressing mutant (P301L) human tau. J. Neurocytol. 32, 1091–1105. 10.1023/B:NEUR.0000021904.61387.95 15044841

[B110] Lin W. L. Zehr C. Lewis J. Hutton M. Yen S. H. Dickson D. W. (2005). Progressive white matter pathology in the spinal cord of transgenic mice expressing mutant (P301L) human tau. J. Neurocytol. 34, 397–410. 10.1007/s11068-006-8726-0 16902761

[B111] Ling H. Hardy J. Zetterberg H. (2015). Neurological consequences of traumatic brain injuries in sports. Mol. Cell. Neurosci. 66, 114–122. 10.1016/j.mcn.2015.03.012 25770439

[B112] Lingor P. Koch J. C. Tonges L. Bahr M. (2012). Axonal degeneration as a therapeutic target in the CNS. Cell Tissue Res. 349, 289–311. 10.1007/s00441-012-1362-3 22392734 PMC3375418

[B113] Lippa C. F. (2004). Synaptophysin immunoreactivity in Pick's disease: comparison with Alzheimer's disease and dementia with Lewy bodies. Am. J. Alzheimers. Dis. Other Demen. 19, 341–344. 10.1177/153331750401900606 15633942 PMC10833996

[B114] Lippa C. F. Zhukareva V. Kawarai T. Uryu K. Shafiq M. Nee L. E.. (2000). Frontotemporal dementia with novel tau pathology and a Glu342Val tau mutation. Ann. Neurol. 48, 850–858. 10.1002/1531-8249(200012)48:6<850::AID-ANA5>3.0.CO;2-V 11117541

[B115] Liu C. Song X. Nisbet R. Gotz J. (2016). Co-immunoprecipitation with Tau Isoform-specific antibodies reveals distinct protein interactions and highlights a putative role for 2N tau in disease. J. Biol. Chem. 291, 8173–8188. 10.1074/jbc.M115.641902 26861879 PMC4825019

[B116] Liu C. W. Lee G. Jay D. G. (1999). Tau is required for neurite outgrowth and growth cone motility of chick sensory neurons. Cell Motil. Cytoskeleton 43, 232–242. 10.1002/(SICI)1097-0169(1999)43:3<232::AID-CM6>3.0.CO;2-7 10401579

[B117] Loomis P. A. Howard T. H. Castleberry R. P. Binder L. I. (1990). Identification of nuclear tau isoforms in human neuroblastoma cells. Proc. Natl. Acad. Sci. U.S.A. 87, 8422–8426. 10.1073/pnas.87.21.8422 1700432 PMC54968

[B118] Ludvigson A. E. Luebke J. I. Lewis J. Peters A. (2011). Structural abnormalities in the cortex of the rTg4510 mouse model of tauopathy: a light and electron microscopy study. Brain Struct. Funct. 216, 31–42. 10.1007/s00429-010-0295-4 21152933 PMC3748379

[B119] Mandelkow E. M. Stamer K. Vogel R. Thies E. Mandelkow E. (2003). Clogging of axons by tau, inhibition of axonal traffic and starvation of synapses. Neurobiol. Aging 24, 1079–1085. 10.1016/j.neurobiolaging.2003.04.007 14643379

[B120] Masters C. L. Bateman R. Blennow K. Rowe C. C. Sperling R. A. Cummings J. L. (2015). Alzheimer's disease. Nat. Rev. Dis. Primers 1:15056 10.1038/nrdp.2015.56 27188934

[B121] Matamoros A. J. Baas P. W. (2016). Microtubules in health and degenerative disease of the nervous system. Brain Res. Bull. 126, 217–225. 10.1016/j.brainresbull.2016.06.016 27365230 PMC5079814

[B122] Maxwell W. L. Mccreath B. J. Graham D. I. Gennarelli T. A. (1995). Cytochemical evidence for redistribution of membrane pump calcium-ATPase and ecto-Ca-ATPase activity, and calcium influx in myelinated nerve fibres of the optic nerve after stretch injury. J. Neurocytol. 24, 925–942. 10.1007/BF01215643 8719820

[B123] Mayford M. Bach M. E. Huang Y. Y. Wang L. Hawkins R. D. Kandel E. R. (1996). Control of memory formation through regulated expression of a CaMKII transgene. Science 274, 1678–1683. 10.1126/science.274.5293.1678 8939850

[B124] McAllister T. W. Ford J. C. Flashman L. A. Maerlender A. Greenwald R. M. Beckwith J. G.. (2014). Effect of head impacts on diffusivity measures in a cohort of collegiate contact sport athletes. Neurology 82, 63–69. 10.1212/01.wnl.0000438220.16190.42 24336143 PMC3873621

[B125] Mckee A. C. Cantu R. C. Nowinski C. J. Hedley-Whyte E. T. Gavett B. E. Budson A. E.. (2009). Chronic traumatic encephalopathy in athletes: progressive tauopathy after repetitive head injury. J. Neuropathol. Exp. Neurol. 68, 709–735. 10.1097/NEN.0b013e3181a9d503 19535999 PMC2945234

[B126] Mckee A. C. Stein T. D. Nowinski C. J. Stern R. A. Daneshvar D. H. Alvarez V. E.. (2012). The spectrum of disease in chronic traumatic encephalopathy. Brain 136(Pt 1), 43–64. 10.1093/brain/aws307 23208308 PMC3624697

[B127] Mckhann G. M. Albert M. S. Grossman M. Miller B. Dickson D. Trojanowski J. Q.. (2001). Clinical and pathological diagnosis of frontotemporal dementia: report of the work group on frontotemporal dementia and pick's disease. Arch. Neurol. 58, 1803–1809. 10.1001/archneur.58.11.1803 11708987

[B128] Meerschaert M. M. Magin R. L. Ye A. Q. (2016). Anisotropic fractional diffusion tensor imaging. J. Vib. Control 22, 2211–2221. 10.1177/1077546314568696 27499605 PMC4973862

[B129] Moreno H. Morfini G. Buitrago L. Ujlaki G. Choi S. Yu E.. (2016). Tau pathology-mediated presynaptic dysfunction. Neuroscience 325, 30–38. 10.1016/j.neuroscience.2016.03.044 27012611 PMC4887082

[B130] Morfini G. A. Burns M. Binder L. I. Kanaan N. M. Lapointe N. Bosco D. A.. (2009). Axonal transport defects in neurodegenerative diseases. J. Neurosci. 29, 12776–12786. 10.1523/JNEUROSCI.3463-09.2009 19828789 PMC2801051

[B131] Morfini G. Pigino G. Beffert U. Busciglio J. Brady S. T. (2002a). Fast axonal transport misregulation and Alzheimer's disease. Neuromolecular Med. 2, 89–99. 10.1385/NMM:2:2:089 12428805

[B132] Morfini G. Pigino G. Mizuno N. Kikkawa M. Brady S. T. (2007). Tau binding to microtubules does not directly affect microtubule-based vesicle motility. J. Neurosci. Res. 85, 2620–2630. 10.1002/jnr.21154 17265463

[B133] Morfini G. Schmidt N. Weissmann C. Pigino G. Kins S. (2016). Conventional kinesin: biochemical heterogeneity and functional implications in health and disease. Brain Res. Bull. 126, 347–353. 10.1016/j.brainresbull.2016.06.009 27339812

[B134] Morfini G. Szebenyi G. Brown H. Pant H. C. Pigino G. Deboer S.. (2004). A novel CDK5-dependent pathway for regulating GSK3 activity and kinesin-driven motility in neurons. EMBO J. 23, 2235–2245. 10.1038/sj.emboj.7600237 15152189 PMC419914

[B135] Morfini G. Szebenyi G. Elluru R. Ratner N. Brady S. T. (2002b). Glycogen synthase kinase 3 phosphorylates kinesin light chains and negatively regulates kinesin-based motility. EMBO J. 21, 281–293. 10.1093/emboj/21.3.281 11823421 PMC125832

[B136] Morfini G. Szebenyi G. Richards B. Brady S. T. (2001). Regulation of kinesin: implications for neuronal development. Dev. Neurosci. 23, 364–376. 10.1159/000048720 11756752

[B137] Morris M. Koyama A. Masliah E. Mucke L. (2011). Tau reduction does not prevent motor deficits in two mouse models of Parkinson's disease. PLoS ONE 6:e29257 10.1371/journal.pone.0029257 22206005 PMC3242771

[B138] Mufson E. J. Perez S. E. Nadeem M. Mahady L. Kanaan N. M. Abrahamson E. E.. (2016). Progression of tau pathology within cholinergic nucleus basalis neurons in chronic traumatic encephalopathy: a chronic effects of neurotrauma consortium study. Brain Inj. 30, 1399–1413. 10.1080/02699052.2016.1219058 27834536 PMC5348250

[B139] Murrell J. R. Spillantini M. G. Zolo P. Guazzelli M. Smith M. J. Hasegawa M.. (1999). Tau gene mutation G389R causes a tauopathy with abundant pick body-like inclusions and axonal deposits. J. Neuropathol. Exp. Neurol. 58, 1207–1226. 10.1097/00005072-199912000-00002 10604746

[B140] Nakamura Y. Takeda M. Yoshimi K. Hattori H. Hariguchi S. Hashimoto S.. (1994). Involvement of clathrin light chains in the pathology of Pick's disease; implication for impairment of axonal transport. Neurosci. Lett. 180, 25–28. 10.1016/0304-3940(94)90905-9 7533277

[B141] Niewidok B. Igaev M. Sundermann F. Janning D. Bakota L. Brandt R. (2016). Presence of a carboxy-terminal pseudorepeat and disease-like pseudohyperphosphorylation critically influence tau's interaction with microtubules in axon-like processes. Mol. Biol. Cell 27, 3537–3549. 10.1091/mbc.E16-06-0402 27582388 PMC5221586

[B142] Padovani A. Borroni B. Brambati S. M. Agosti C. Broli M. Alonso R.. (2006). Diffusion tensor imaging and voxel based morphometry study in early progressive supranuclear palsy. J. Neurol. Neurosurg. Psychiatr. 77, 457–463. 10.1136/jnnp.2005.075713 16306152 PMC2077489

[B143] Papasozomenos S. C. Binder L. I. (1987). Phosphorylation determines two distinct species of Tau in the central nervous system. Cell Motil. Cytoskeleton 8, 210–226. 10.1002/cm.970080303 2446784

[B144] Petrie E. C. Cross D. J. Yarnykh V. L. Richards T. Martin N. M. Pagulayan K.. (2014). Neuroimaging, behavioral, and psychological sequelae of repetitive combined blast/impact mild traumatic brain injury in Iraq and Afghanistan war veterans. J. Neurotrauma 31, 425–436. 10.1089/neu.2013.2952 24102309 PMC3934596

[B145] Pollock N. J. Mirra S. S. Binder L. I. Hansen L. A. Wood J. G. (1986). Filamentous aggregates in Pick's disease, progressive supranuclear palsy, and Alzheimer's disease share antigenic determinants with microtubule-associated protein, tau. Lancet 2:1211. 10.1016/S0140-6736(86)92212-9 2430155

[B146] Praprotnik D. Smith M. A. Richey P. L. Vinters H. V. Perry G. (1996). Filament heterogeneity within the dystrophic neurites of senile plaques suggests blockage of fast axonal transport in Alzheimer's disease. Acta Neuropathol. 91, 226–235. 10.1007/s004010050420 8834534

[B147] Probst A. Gotz J. Wiederhold K. H. Tolnay M. Mistl C. Jaton A. L.. (2000). Axonopathy and amyotrophy in mice transgenic for human four-repeat tau protein. Acta Neuropathol. 99, 469–481. 10.1007/s004010051148 10805089

[B148] Probst A. Langui D. Lautenschlager C. Ulrich J. Brion J. P. Anderton B. H. (1988). Progressive supranuclear palsy: extensive neuropil threads in addition to neurofibrillary tangles. Very similar antigenicity of subcortical neuronal pathology in progressive supranuclear palsy and Alzheimer's disease. Acta Neuropathol. 77, 61–68. 10.1007/BF00688244 3149122

[B149] Probst A. Tolnay M. Langui D. Goedert M. Spillantini M. G. (1996). Pick's disease: hyperphosphorylated tau protein segregates to the somatoaxonal compartment. Acta Neuropathol. 92, 588–596. 10.1007/s004010050565 8960316

[B150] Qiang L. Yu W. Andreadis A. Luo M. Baas P. W. (2006). Tau protects microtubules in the axon from severing by katanin. J. Neurosci. 26, 3120–3129. 10.1523/JNEUROSCI.5392-05.2006 16554463 PMC6674103

[B151] Raff M. C. Whitmore A. V. Finn J. T. (2002). Axonal self-destruction and neurodegeneration. Science 296, 868–871. 10.1126/science.1068613 11988563

[B152] Ramirez-Rios S. Denarier E. Prezel E. Vinit A. Stoppin-Mellet V. Devred F.. (2016). Tau antagonizes end-binding protein tracking at microtubule ends through a phosphorylation-dependent mechanism. Mol. Biol. Cell 27, 2924–2934. 10.1091/mbc.E16-01-0029 27466319 PMC5042579

[B153] Rao M. V. Mcbrayer M. K. Campbell J. Kumar A. Hashim A. Sershen H.. (2014). Specific calpain inhibition by calpastatin prevents tauopathy and neurodegeneration and restores normal lifespan in tau P301L mice. J. Neurosci. 34, 9222–9234. 10.1523/JNEUROSCI.1132-14.2014 25009256 PMC4087203

[B154] Rasband M. N. (2010). The axon initial segment and the maintenance of neuronal polarity. Nat. Rev. Neurosci. 11, 552–562. 10.1038/nrn2852 20631711

[B155] Rebeiz J. J. Kolodny E. H. Richardson E. P. Jr. (1968). Corticodentatonigral degeneration with neuronal achromasia. Arch. Neurol. 18, 20–33. 10.1001/archneur.1968.00470310034003 5634369

[B156] Rizzu P. Van Swieten J. C. Joosse M. Hasegawa M. Stevens M. Tibben A.. (1999). High prevalence of mutations in the microtubule-associated protein tau in a population study of frontotemporal dementia in the Netherlands. Am. J. Hum. Genet. 64, 414–421. 10.1086/302256 9973279 PMC1377751

[B157] Rodriguez-Martin T. Pooler A. M. Lau D. H. Morotz G. M. De Vos K. J. Gilley J.. (2016). Reduced number of axonal mitochondria and tau hypophosphorylation in mouse P301L tau knockin neurons. Neurobiol. Dis. 85, 1–10. 10.1016/j.nbd.2015.10.007 26459111 PMC4684147

[B158] Rohrer J. D. Ridgway G. R. Modat M. Ourselin S. Mead S. Fox N. C.. (2010). Distinct profiles of brain atrophy in frontotemporal lobar degeneration caused by progranulin and tau mutations. Neuroimage 53, 1070–1076. 10.1016/j.neuroimage.2009.12.088 20045477 PMC2941039

[B159] Samsonov A. Yu J. Z. Rasenick M. Popov S. V. (2004). Tau interaction with microtubules *in vivo*. J. Cell Sci. 117, 6129–6141. 10.1242/jcs.01531 15564376

[B160] Santacruz K. Lewis J. Spires T. Paulson J. Kotilinek L. Ingelsson M.. (2005). Tau suppression in a neurodegenerative mouse model improves memory function. Science 309, 476–481. 10.1126/science.1113694 16020737 PMC1574647

[B161] Seitz A. Kojima H. Oiwa K. Mandelkow E. M. Song Y. H. Mandelkow E. (2002). Single-molecule investigation of the interference between kinesin, tau and MAP2c. EMBO J. 21, 4896–4905. 10.1093/emboj/cdf503 12234929 PMC126299

[B162] Selden S. C. Pollard T. D. (1986). Interaction of actin filaments with microtubules is mediated by microtubule-associated proteins and regulated by phosphorylation. Ann. N. Y. Acad. Sci. 466, 803–812. 10.1111/j.1749-6632.1986.tb38464.x 3460455

[B163] Siman R. Cocca R. Dong Y. (2015). The mTOR inhibitor rapamycin mitigates perforant pathway neurodegeneration and synapse loss in a mouse model of early-stage alzheimer-type tauopathy. PLoS ONE 10:e0142340. 10.1371/journal.pone.0142340 26540269 PMC4634963

[B164] Siman R. Lin Y. G. Malthankar-Phatak G. Dong Y. (2013). A rapid gene delivery-based mouse model for early-stage Alzheimer disease-type tauopathy. J. Neuropathol. Exp. Neurol. 72, 1062–1071. 10.1097/NEN.0000000000000006 24128676 PMC3815088

[B165] Sjobeck M. Englund E. (2003). Glial levels determine severity of white matter disease in Alzheimer's disease: a neuropathological study of glial changes. Neuropathol. Appl. Neurobiol. 29, 159–169. 10.1046/j.1365-2990.2003.00456.x 12662323

[B166] Sjobeck M. Haglund M. Englund E. (2005). Decreasing myelin density reflected increasing white matter pathology in Alzheimer's disease–a neuropathological study. Int. J. Geriatr. Psychiatry 20, 919–926. 10.1002/gps.1384 16163742

[B167] Song Y. Brady S. T. (2015). Post-translational modifications of tubulin: pathways to functional diversity of microtubules. Trends Cell Biol. 25, 125–136. 10.1016/j.tcb.2014.10.004 25468068 PMC4344850

[B168] Spires T. L. Orne J. D. Santacruz K. Pitstick R. Carlson G. A. Ashe K. H.. (2006). Region-specific dissociation of neuronal loss and neurofibrillary pathology in a mouse model of tauopathy. Am. J. Pathol. 168, 1598–1607. 10.2353/ajpath.2006.050840 16651626 PMC1606598

[B169] Spittaels K. Van Den Haute C. Van Dorpe J. Bruynseels K. Vandezande K. Laenen I.. (1999). Prominent axonopathy in the brain and spinal cord of transgenic mice overexpressing four-repeat human tau protein. Am. J. Pathol. 155, 2153–2165. 10.1016/S0002-9440(10)65533-2 10595944 PMC1866931

[B170] Stamer K. Vogel R. Thies E. Mandelkow E. Mandelkow E. M. (2002). Tau blocks traffic of organelles, neurofilaments, and APP vesicles in neurons and enhances oxidative stress. J. Cell Biol. 156, 1051–1063. 10.1083/jcb.200108057 11901170 PMC2173473

[B171] Stern J. L. Lessard D. V. Hoeprich G. J. Morfini G. A. Berger C. L. (2017). Phospho-regulation of tau modulates inhibition of kinesin-1 motility. Mol. Biol. Cell. 28, 1079–1087. 10.1091/mbc.E16-10-0728 28251926 PMC5391184

[B172] Stoub T. R. Detoledo-Morrell L. Stebbins G. T. Leurgans S. Bennett D. A. Shah R. C. (2006). Hippocampal disconnection contributes to memory dysfunction in individuals at risk for Alzheimer's disease. Proc. Natl. Acad. Sci. U.S.A. 103, 10041–10045. 10.1073/pnas.0603414103 16785436 PMC1479867

[B173] Sudo H. Baas P. W. (2011). Strategies for diminishing katanin-based loss of microtubules in tauopathic neurodegenerative diseases. Hum. Mol. Genet. 20, 763–778. 10.1093/hmg/ddq521 21118899 PMC3024046

[B174] Sultan A. Nesslany F. Violet M. Begard S. Loyens A. Talahari S.. (2011). Nuclear tau, a key player in neuronal DNA protection. J. Biol. Chem. 286, 4566–4575. 10.1074/jbc.M110.199976 21131359 PMC3039398

[B175] Sun W. Qureshi H. Y. Cafferty P. W. Sobue K. Agarwal-Mawal A. Neufield K. D.. (2002). Glycogen synthase kinase-3beta is complexed with tau protein in brain microtubules. J. Biol. Chem. 277, 11933–11940. 10.1074/jbc.M107182200 11812770

[B176] Swanson E. Breckenridge L. Mcmahon L. Som S. Mcconnell I. Bloom G. S. (2017). Extracellular tau oligomers induce invasion of endogenous tau into the somatodendritic compartment and axonal transport dysfunction. J. Alzheimers. Dis. 58, 803–820. 10.3233/JAD-170168 28482642 PMC5581403

[B177] Takei Y. Teng J. Harada A. Hirokawa N. (2000). Defects in axonal elongation and neuronal migration in mice with disrupted tau and map1b genes. J. Cell Biol. 150, 989–1000. 10.1083/jcb.150.5.989 10973990 PMC2175245

[B178] Terry R. D. Masliah E. Salmon D. P. Butters N. Deteresa R. Hill R.. (1991). Physical basis of cognitive alterations in Alzheimer's disease: synapse loss is the major correlate of cognitive impairment. Ann. Neurol. 30, 572–580. 10.1002/ana.410300410 1789684

[B179] Tiernan C. T. Combs B. Cox K. Morfini G. Brady S. T. Counts S. E.. (2016). Pseudophosphorylation of tau at S422 enhances SDS-stable dimer formation and impairs both anterograde and retrograde fast axonal transport. Exp. Neurol. 283, 318–329. 10.1016/j.expneurol.2016.06.030 27373205 PMC4992631

[B180] Tint I. Slaughter T. Fischer I. Black M. M. (1998). Acute inactivation of tau has no effect on dynamics of microtubules in growing axons of cultured sympathetic neurons. J. Neurosci. 18, 8660–8673. 9786973 10.1523/JNEUROSCI.18-21-08660.1998 PMC6793543

[B181] Tokuda T. Ikeda S. Yanagisawa N. Ihara Y. Glenner G. G. (1991). Re-examination of ex-boxers' brains using immunohistochemistry with antibodies to amyloid beta-protein and tau protein. Acta Neuropathol. 82, 280–285. 10.1007/BF00308813 1759560

[B182] Tucker K. L. Meyer M. Barde Y. A. (2001). Neurotrophins are required for nerve growth during development. Nat. Neurosci. 4, 29–37. 10.1038/82868 11135642

[B183] Uryu K. Chen X. H. Martinez D. Browne K. D. Johnson V. E. Graham D. I.. (2007). Multiple proteins implicated in neurodegenerative diseases accumulate in axons after brain trauma in humans. Exp. Neurol. 208, 185–192. 10.1016/j.expneurol.2007.06.018 17826768 PMC3979356

[B184] Uversky V. N. (2015). Intrinsically disordered proteins and their (disordered) proteomes in neurodegenerative disorders. Front. Aging Neurosci. 7:18. 10.3389/fnagi.2015.00018 25784874 PMC4345837

[B185] Vanderweyde T. Apicco D. J. Youmans-Kidder K. Ash P. E. Cook C. Lummertz Da Rocha E.. (2016). Interaction of tau with the RNA-Binding Protein TIA1 regulates tau pathophysiology and toxicity. Cell Rep. 15, 1455–1466. 10.1016/j.celrep.2016.04.045 27160897 PMC5325702

[B186] van Hummel A. Bi M. Ippati S. Van Der Hoven J. Volkerling A. Lee W. S.. (2016). No overt deficits in aged tau-deficient C57Bl/6.Mapttm1(EGFP)Kit GFP knockin mice. PLoS ONE 11:e0163236. 10.1371/journal.pone.0163236 27736877 PMC5063411

[B187] Vermersch P. Roche J. Hamon M. Daems-Monpeurt C. Pruvo J. P. Dewailly P.. (1996). White matter magnetic resonance imaging hyperintensity in Alzheimer's disease: correlations with corpus callosum atrophy. J. Neurol. 243, 231–234. 10.1007/BF00868519 8936352

[B188] Vossel K. A. Xu J. C. Fomenko V. Miyamoto T. Suberbielle E. Knox J. A.. (2015). Tau reduction prevents Abeta-induced axonal transport deficits by blocking activation of GSK3beta. J. Cell Biol. 209, 419–433. 10.1083/jcb.201407065 25963821 PMC4427789

[B189] Walsh D. M. Selkoe D. J. (2016). A critical appraisal of the pathogenic protein spread hypothesis of neurodegeneration. Nat. Rev. Neurosci. 17, 251–260. 10.1038/nrn.2016.13 26988744 PMC6701169

[B190] Wang L. N. Zhu M. W. Feng Y. Q. Wang J. H. (2006). Pick's disease with Pick bodies combined with progressive supranuclear palsy without tuft-shaped astrocytes: a clinical, neuroradiologic and pathological study of an autopsied case. Neuropathology 26, 222–230. 10.1111/j.1440-1789.2006.00671.x 16771179

[B191] Weingarten M. D. Lockwood A. H. Hwo S. Y. Kirschner M. W. (1975). A protein factor essential for microtubule assembly. Proc. Natl. Acad. Sci. U.S.A. 72, 1858–1862. 10.1073/pnas.72.5.1858 1057175 PMC432646

[B192] Werth J. L. Thayer S. A. (1994). Mitochondria buffer physiological calcium loads in cultured rat dorsal root ganglion neurons. J. Neurosci. 14, 348–356. 8283242 10.1523/JNEUROSCI.14-01-00348.1994 PMC6576848

[B193] Whitwell J. L. Master A. V. Avula R. Kantarci K. Eggers S. D. Edmonson H. A.. (2011). Clinical correlates of white matter tract degeneration in progressive supranuclear palsy. Arch. Neurol. 68, 753–760. 10.1001/archneurol.2011.107 21670399 PMC3401587

[B194] Williams D. R. Lees A. J. (2009). Progressive supranuclear palsy: clinicopathological concepts and diagnostic challenges. Lancet Neurol. 8, 270–279. 10.1016/S1474-4422(09)70042-0 19233037

[B195] Wszolek Z. K. Pfeiffer R. F. Bhatt M. H. Schelper R. L. Cordes M. Snow B. J.. (1992). Rapidly progressive autosomal dominant parkinsonism and dementia with pallido-ponto-nigral degeneration. Ann. Neurol. 32, 312–320. 10.1002/ana.410320303 1416801

[B196] Xia D. Li C. Gotz J. (2015). Pseudophosphorylation of Tau at distinct epitopes or the presence of the P301L mutation targets the microtubule-associated protein Tau to dendritic spines. Biochim. Biophys. Acta 1852, 913–924. 10.1016/j.bbadis.2014.12.017 25558816

[B197] Yamakawa K. Takanashi M. Watanabe M. Nakamura N. Kobayashi T. Hasegawa M.. (2006). Pathological and biochemical studies on a case of Pick disease with severe white matter atrophy. Neuropathology 26, 586–591. 10.1111/j.1440-1789.2006.00738.x 17203597

[B198] Yasuda M. Mayford M. R. (2006). CaMKII activation in the entorhinal cortex disrupts previously encoded spatial memory. Neuron 50, 309–318. 10.1016/j.neuron.2006.03.035 16630840

[B199] Yin Y. Wang Y. Gao D. Ye J. Wang X. Fang L.. (2016). Accumulation of human full-length tau induces degradation of nicotinic acetylcholine receptor alpha4 via activating calpain-2. Sci. Rep. 6:27283. 10.1038/srep27283 27277673 PMC4899694

[B200] Yoshiyama Y. Zhang B. Bruce J. Trojanowski J. Q. Lee V. M. (2003). Reduction of detyrosinated microtubules and Golgi fragmentation are linked to tau-induced degeneration in astrocytes. J. Neurosci. 23, 10662–10671. 14627651 10.1523/JNEUROSCI.23-33-10662.2003 PMC6740917

[B201] Yuan A. Kumar A. Peterhoff C. Duff K. Nixon R. A. (2008). Axonal transport rates *in vivo* are unaffected by tau deletion or overexpression in mice. J. Neurosci. 28, 1682–1687. 10.1523/JNEUROSCI.5242-07.2008 18272688 PMC2814454

[B202] Zhang L. Ravdin L. D. Relkin N. Zimmerman R. D. Jordan B. Lathan W. E.. (2003). Increased diffusion in the brain of professional boxers: a preclinical sign of traumatic brain injury? AJNR Am. J. Neuroradiol. 24, 52–57. 12533327 PMC8148951

[B203] Zhang Y. Walter R. Ng P. Luong P. N. Dutt S. Heuer H.. (2016). Progression of microstructural degeneration in progressive supranuclear palsy and corticobasal syndrome: a longitudinal diffusion tensor imaging study. PLoS ONE 11:e0157218. 10.1371/journal.pone.0157218 27310132 PMC4911077

[B204] Zhou L. Miller B. L. Mcdaniel C. H. Kelly L. Kim O. J. Miller C. A. (1998). Frontotemporal dementia: neuropil spheroids and presynaptic terminal degeneration. Ann. Neurol. 44, 99–109. 10.1002/ana.410440116 9667597

